# Socio-economic factors related with the subjective well-being of the rural elderly people living independently in China

**DOI:** 10.1186/s12939-015-0136-4

**Published:** 2015-01-17

**Authors:** Yicheng Zhou, Linyi Zhou, Changluan Fu, You Wang, Qingle Liu, Hongtao Wu, Rongjun Zhang, Linfeng Zheng

**Affiliations:** School of Politics and Public Administration, Soochow University, Collaborative Innovation Center for New-type Urbanization and Social Governance of Jiangsu Province, Suzhou, China; School of Stastistics and Mathemetics, Yunnan University of Finance and Economics, Kunming, China; School of Management, Zhejiang International Studies University, Hangzhou, China; School of Philosophy and sociology, Northwest University, Xi′an, China; School of Government, Central University of Finance and Economics, Beijing, China; The first affiliated hospital of Soochow University, Suzhou, China; Nanjing University of Science and Technology, Nanjing, China; Chinese Academy of Sciences, Nanjing, China

**Keywords:** MUNSH, SWB, The elderly living independently, Wenzhou, China

## Abstract

**Background:**

Many Chinese elderly increasingly face the serious problem of the “empty nest” phenomenon. The elderly living independently, also called empty-nest elderly, refers to elderly people living alone whose children left home. However few studies concerned about the subjective well-being (SWB) of the elderly living independently.

**Methods:**

This study employs The Memorial University of Newfoundland Scale of Happiness (MUNSH) to explore the SWB of the elderly living independently in rural areas of Wenzhou, a relatively developed region in China. 536 sampled are randomly selected.

**Results:**

The results indicate that participants obtained low scores in positive affect, positive experience, and the total SWB score, but high scores in negative affect and negative experience. Age, low education, poor health condition and little income were found to be negatively correlated with SWB. The SWB score of the elderly living with a spouse is higher than those who divorced or lost their spouse and the score of women is lower than that of men. In addition, the survey revealed that children’s support has a positive influence on the SWB of the rural elderly living independently.

**Conclusions:**

The elderly living independently in rural Wenzhou, China have unfavorable SWB. Poor socio-economic statuses are negative impact factors. But the children’s support can help to improve. Special attention is needed to those with lower socio-economic status and less children’s support.

## Introduction

The sixth national census data in 2010 show that the elderly aged 65 and above account for 8.87%, which is 1.91 percentage points higher than that in 2000. Changes in the age structure of the population indicate that the aging process gradually speeds up [1]. Many Chinese elderly increasingly face the serious problem of the "empty nest", which indicated they live independently or alone. According to the 2013 data by China National Committee On Ageing, the number of China’s empty-nest elderly carried on rising. There were 99 million in 2012 and the number in 2013 would up to 100 million [2]. This phenomenon has become an important issue in the aging process that cannot be ignored. This study will sample the rural elderly living independently in Wenzhou, a relatively developed city in Eastern China, use scientific and reliable scale to measure their subjective well-being (SWB) and explore its socio-economic factors.

### Health conditions of the Chinese elderly living independently

The elderly living independently are people who do not live with their children or have no children. Studies on Chinese rural elderly living independently are relatively extensive. The causes of empty-nest phenomenon are very complicated and multifaceted, including the population migration due to the economic reform, the strict one-child policy, improvement of people’s life standard and the development of healthcare industry and so on [1,3].

Many Chinese scholars have focused particularly on the case study of the pension problem [4,5]. They consider the elderly living independently to have more problems in many aspects, such as disease care, life arrangement, and health, among others, compared with the non-empty-nest elderly. Particularly, studies concerned about their mental health condition, such as depression, loneliness, life satisfaction and quality of life (QOL).

Many elderly living independently experience a high risk of depression. It is indicated that the prevalence of depression in empty-nest elderly is higher than that in non-empty-nest elderly [6]. Some scholars conducted an investigation on the elderly living independently in rural areas and found that the determined prevalence of depression was high at 74.46%, much higher than that of non-empty-nest elderly [7]. Others also explored the symptoms of depression in the elderly living independently in Hunan Province and found that the condition was worse in rural areas [8]. It was reported that 30.11% of the sampled elderly living independently in Sichuan Province showed anxiety or anxiety-related symptoms [9]. The elderly living independently who were more depressed, were female, living in rural areas, and had cognitive impairment were in danger of suffering from depression.

Loneliness is also common among the elderly living independently. It's found that the empty-nest elderly or rural elderly had stronger feelings of loneliness [10,11]. Loneliness was prevalent among the empty-nest elderly in rural Anhui Province [12]. They also had lower physical and psychological health scores, weaker relationships with children and social support; and higher prevalence of chronic diseases than non-empty-nest elderly. The empty-nest elderly are more likely to have mental disorders and lower life satisfaction [13]. The negative psychological phenomenon could be prevented or eased if the elderly living independently could obtain better social support and medical care. The help from the neighbors offer could alleviate their risk being excluded from the society [14].

The health or QOL of the elderly living independently is worrying [15,16]. Data from the national household survey conducted in 2008 and found that the elderly who living alone reported the worse health-related quality of life (HRQOL) compared with that of the non-empty-nest elderly or the elderly who lived with their spouses [17]. The elderly living independently was less likely to consult doctors [18]. This finding indicates the fewer chances for medical care and the lower levels of income and social support of the empty-nest elderly. The QOL of Chinese elderly living independently are found that their scores of vitality, role emotional, and mental health in the mental component summary were lower than those of the non-empty-nest elderly [19].

Above literatures indicate the phenomenon that the elderly living independently have worrying mental health condition. Their distinctive living arrangement risks them on high prevalence of mental disorders. Therefore their mental health needs more special attention. But as we can see, many of the abovementioned literatures studied the negative moods, morbidity, and general health of the elderly. Few study the positive experiences or affects of the elderly from the aspiring perspective. As far as we are concerned, only few literatures explored their well-being, such as the psychological well-being [20]. This study intends to fill the research gap, aiming at studying the SWB of the elderly living independently in China.

### Subjective well-being

SWB is a broad category of phenomena that includes people's emotional responses, domain satisfactions, and global judgments of life satisfaction [21]. SWB has three components: life satisfaction, pleasant affect, and unpleasant affect. The reason why measuring SWB is popular is that that it can depend on one’s own opinion to self-rate his / her feelings, which complies with the worldwide epidemic of individualism [22]. SWB is affected by many factors, such as health, income, religion, marriage, age, gender, occupation, and education, among others. As a scientific observation, SWB has been applied for an index to measure people’s satisfaction or happiness in a nation [22,23].

Studies focused on the Chinese elderly show that living alone is associated with low SWB, and living with their immediate families is associated with positive SWB. Living in rural areas or alone is related to low life satisfaction and unpleasant emotional experience [24]. Family support and cognitive function are key factors to their QOL [25]. It is reported that social support and negative interactions could significantly affect the SWB of the Chinese elderly and lead to a depressive effect [26]. An investigation in a relatively poor region in Hunan Province and found that education, income, and social support had unique and significant impacts on SWB [27]. Above literatures focused on the SWB condition or their factors of the Chinese elderly but not the empty-nest elderly or the elderly living independently.

### Research hypotheses

SWB is affected by many factors [28]. Among them, socio-demographic variables such as gender, age and so on are thought to be very important factors [29]. The following is our first hypothesis:

Hypothesis 1: The SWB of the Chinese rural elderly living independently has differences in terms of demographic variables (i.e., gender, age, and education).

China's traditional family model is made up of three generations live under one roof. It is considered this traditional model to be beneficial to the elderly in rural areas because it represents the fulfillment of a cultural yearning [20]. The elderly who live either with their children and grandchildren or with their grandchildren had stronger feelings than those who live alone. Financial support from their children or harmonious family relations can enhance SWB. Rural grandparents with filial children and harmonious families have high life satisfaction [30]. Support between generations can be beneficial to the psychological health of the elderly [31]. It is indicated that the loneliness of the oldest old decreased with the presence of their children or the love of children [32]. The feeling of fondness can be increased by the elderly keeping in touch with their children. Therefore, our second hypothesis is as follow:

Hypothesis 2: Mental support from the children can enhance the SWB of the Chinese rural elderly living independently.

### Research objectives

The Chinese social pension system, especially the social endowment care in rural areas, is far from effective. The elderly living independently face uncertainties in many aspects such as economic support, social care and spiritual solace among others. Therefore the elderly living independently is therefore not only an issue related to specific families but also to the overall society. The empty-nest elderly issue is associated with the improvement of people’s QOL and the long-term development of social harmony and stability. Studying the SWB of the elderly living independently has theoretical and practical significance.

This study explores the SWB of the elderly living independently in the rural areas of Wenzhou, a relatively developed region in China. The study aims to provide evidence on the SWB of the Chinese rural empty-nest elderly and to give suggestions to the government or social organizations on how to improve the SWB of the elderly living independently. It also hopes to provide a reference value for work on other parts of China and to further arouse the society’s concern about the SWB of the elderly living independently.

## Methods

### Sample and procedures

The data used in this article originated from the sample of the elderly living independently aged 65 to 95 interviewed by the Research Center for Wenzhouese Economy. The elderly living independently were defined as people who do not always live with any of their children and those who have no children. Wenzhou is the capital city of the economic zone on the west side of the straits. Wenzhou has absorbed the most number of migrants from Zhejiang Province over the years. But in the meantime, the trend of ageing and empty-nesting are also accelerating. By 2015, the number of the elderly aged 60 in Wenzhou will grow up from 1.0076 million to 1.2689 million, proposing greater challenges to the social security industry for the elderly [33].

The multi-stage stratified cluster sampling method is mainly used in this study. *First,* each region was randomly selected from three districts (Lucheng, Longwan, and Ouhai), two cities (Rui’an and Yueqing), and six counties (Dongtou, Yongjia, Pingyang, Cangnan, Taishun and Wencheng). Three regions were then selected: Longwan District, Yueqing City, and Dongtou County. *Second,* two villages were randomly selected. All the elderly aged 65 to 95 in each village were taken as research objects. Only one elderly was interviewed in each household. A total of 536 (99.63%) valid questionnaires were collected from the male and female participants.

### Measures

This study explored the SWB of the rural elderly living independently aged 65 to 95 who lived in Wenzhou City. Each questionnaire was conducted through face-to-face interviews between a trained social worker and the respondents. The investigator read every item in the questionnaire in a neutral, unbiased manner as most of the respondents were old and had relatively low education. The investigator filled out the questionnaire accordingly after ensuring that the respondents understood the question and gave their answers independently. The questionnaire has two components: basic information and the SWB scale.

1. Basic information includes age, gender, marriage, education, number of children, self-rated health, income, relationship with their children, and social relationship, among others.
2. Memorial University of Newfoundland Scale of Happiness (MUNSH).

There are many good measurements to measure SWB. The simplest one is the single item asking the respondents how happy or satisfied they are [34]. The popular and common ones are the Satisfaction With Life Scale (SWLS) [35], the Positive and Negative Affect Schedule (PANAS). [36] and so on. But only MUNSH is particularly suitable for the elderly people. Many of its items are directed to the old [28,37].

Kozma and Stones [38] developed the MUNSH based on the “life satisfaction index” and emotional balance scale put forward by Bradburn and Beise [39], respectively. This scale was first applied to the elderly aged 65 to 95 living in urban and rural areas and in elderly apartments in Newfoundland. Its validity and reliability were higher than those of previous scales used to measure SWB. The re-validation was also confirmed [40]. The MUNSH has been used since then in many countries to measure the mental health condition of the elderly, including Chinese older adults [41].

MUNSH consists of 24 items: 5 items indicate positive affect (PA), 5 items reflect negative affect (NA), 7 items indicate positive experience (PE), and 7 items indicate negative experience (NE). The answer “yes” is coded as 2 points, the answer “don’t know” is coded as 1 point, and the answer “no” is coded as 0 point for each item. The 19^th^ item presents the answer choices for “present living place”, which is coded as 2 points, and “other living places”, which is coded as 0 point. The 23^rd^ item presents the answer choices for “satisfied”, which is coded as 2 points, and “not satisfied,” which is coded as 0 point. The total score is computed b by the following formula: SWB = PA-NA + PE-NE. The constant “24” is always added for the convenience of calculation. The scores ranged from 0 to 48 points. The higher the score is, the happier the respondent. The following are the criteria: when the score is 36 or above, the SWB is at a high level; when the score is 12 or below, the SWB is at a low level; and when the score ranges from 12 to 36, the SWB is at the middle level.

### Ethical statement

The survey conducted with oral informed consent and the approval of the ethics committee of the university, in compliance with the principles of the Declaration of Helsinki. Interviewers informed each respondent of their right to refuse to participate, and of their right to refuse to answer any question, both initially and during the course of the research.

## Results

### Descriptive statistics

The NA and NE were considered to be negative before the validity and reliability of the scale are tested because the MUNSH includes both positive and negative experiences. Reliability tests were conducted on the 24 items of the four domains of the MUNSH. The Cronbach’s alpha coefficient of the whole scale was 0.823, which indicates that the questionnaire had good reliability. The Cronbach’s alpha coefficients of the four domains were 0.761, 0.782, 0.754, and 0.821, respectively, which indicate high reliability. Item internal consistency and item discriminant validity were analyzed to ensure the validity effect of the scale. The Pearson correlation coefficient was calculated among the 24 items and their corresponding domains as well as the other three domains. The correlation coefficient between items and its domain should eliminate duplication. Only the correlation coefficient between the item’s score and that of other items that belong to the same domain was calculated. When the correlation coefficient between the item and its domain was equal to or larger than 0.40, one successful convergent validity test was recorded. When the correlation coefficient between the item and its domain was significantly higher than that of other domains, one successful discriminant validity test was recorded. The results showed that the correlation coefficients of all items and their hypothetical domains were larger than 0.40_._ The 24 convergent validity tests were all successful with a success rate of 100%. Out of the 72 discriminant validity tests, 65 were successful with a success rate of 90.23%. Overall, the validity tests were good.

A total of 538 questionnaires were distributed, and 536 effective ones were received. The effective rate was 99.62%. In investigating the SWB of 536 rural elderly living independently, we found that they obtained low scores in PA (2.66), PE (4.23), and total SWB score (15.81); they obtained high scores in NA (6.47) and NE (8.60).

We scored the participants on the following variables: gender, marriage, education, number of children, self-rated health condition, income, relationships with children, social relationship among others. Variance analysis was conducted on the SWB of the rural elderly living independently using different factors.

The rural elderly living independently was composed of 256 males and 280 females, as indicated in Table 1. The average age was 76.23 ± 7.86 years old. Among the participants, 265 respondents were aged between 65 and 75 years old, 198 respondents aged between 75 and 85 years old, and 73 respondents aged between 85 and 95 years old. A total of 432 respondents had primary school and below education, 79 had middle school education, and 25 had high school and above education. A total of 385 were married and had spouses; 151 of them were divorced, widowed or unmarried. Besides, 132 elderly reported good health while 203 and 201 ones reported fair and poor health conditions. Most of the elderly have 2 and more children, fair or low income. And 61% of the respondents have rare relationship with their children. The SWB of these rural empty-nest elderly had statistically significant differences in terms of gender, age, marriage, education, self-rated health condition, number of children, income, and relationships with children, among others (*P* <0.01).

**Table 1:** **Variance analysis of the basic information and the SWB with difference factors**

**Variables**	**Definition**	**N (people)**	**Mean value**	**standard deviation**	***P*** **-value**
Age	65–75 years old	265	15.97	3.13	0.0000
	75–85 years old	198	15.83	3.12	
	85–95 years old	73	15.19	3.09	
Gender	Male	256	15.62	3.10	0.0000
	Female	280	15.99	3.14	
Marriage	Married have spouses	385	16.21	3.15	0.0000
	Divorced, widowed, or unmarried	151	15.66	3.03	
Education	Primary school and below	432	15.84	3.18	0.0000
	Middle school	79	15.97	2.75	
	High school and above	25	14.72	3.12	
Self-rated health condition	good	132	16.11	3.06	0.0000
	fair	203	15.66	3.22	
	poor	201	15.77	3.07	
Number of children	0	14	15.50	2.77	0.0000
	1–2	156	15.65	3.15	
	2 and above	366	15.89	3.13	
Income	Good	69	16.25	3.15	0.0000
	Fair	344	15.77	3.16	
	Poor	123	15.69	3.02	
Relationship with children	Frequent	86	15.63	3.35	0.0000
	Sometimes	123	15.92	3.06	
	Rare	327	15.82	3.09	

### Impact factor of the SWB of rural elderly living independently

This study used a logistic model to analyze the impact factors of the Chinese rural elderly living independently. The logistic model used is the regression model that focuses on the two-categorical or multi- categorical dependent variables. Explanatory variables can be nominal data or quantitative data. As the SWB in this study was an attitude-orientation sentiment classification question with certain orders, a logistics model called proportion odds model with ordinal categorical explanatory variable was used to fully use and reflect this relationship. Hypothetically, the format of the dependent variable *Y* and its value in the proportional odds model is

$$ \log\;it\left(P\left(Y\le j\right)\right)={\beta}_0+{\beta}_1{X}_1+\cdot \cdot \cdot +{\beta}_p{X}_p, $$

where

$$ \log\;it\left(P\left(Y\le j\right)\right)=1\mathrm{n}\left(\raisebox{1ex}{$P\left(Y\le j\right)$}\!\left/ \!\raisebox{-1ex}{$1-P\left(Y\le j\right)$}\right.\right) $$

An important application of the logistic model is the estimated odds ratio (OR). OR is the ratio of the occurrence probability over the non-occurrence probability of certain events. It is used to analyze the advantages caused by the change in one explanatory variable and the effect of dependent variables. The OR of proportional odds model is

$$ OR=\frac{P\left({Y}_i\le j\right)/P\left({Y}_i>j\right)}{P\left({Y}_j\le j\right)/P\left({Y}_j>j\right).} $$

The unknown population parameter estimation of the model commonly uses the maximum likelihood estimation methods because the logistic regression model is nonlinear. The estimation results have consistency, asymptotic efficiency, and asymptotic normality. The null hypothesis of the parameter estimation of the logistic regression model can be tested through the chi-square of likelihood ratio, score, and Wald tests. The goodness of fit of the model can be determined by the deviance and Pearson criteria. The parameter estimation and test process were conducted using SAS 9.2.

This study used the scores of the MUNSH scale to determine the SWB of the respondents. The SWB scores < = 12 indicate unhappy, 12 < SWB scores of < 36 indicate fairly happy, and SWB scores > 36 indicate happy. Gender, marriage, education, number of children, self-rated health, income, relationship with their children, and social relationship, among others, were used as the independent variables.

The SWB of the rural elderly living independently is a three-categorical dependent variable: unhappy = 1, fairly happy = 2, and happy = 3. Therefore, the format of the established proportional odds model is

$$ \log it\left({P}_1\right)=1\mathrm{n}\left(\frac{P_1}{1-{P}_1}\right)={\alpha}_1+\beta \hbox{'}X+{\varepsilon}_i, $$

$$ \log it\left({P}_1+{P}_2\right)=1\mathrm{n}\left(\frac{P_1+{P}_2}{P_3}\right)={\alpha}_2+\beta \hbox{'}+{\varepsilon}_i, $$

where *α*_1_ and *α*_2_ are constant terms, and *β* ∈ *R* is the regression estimation parameter vector. *P*_1_, *P*_2__,_ and *P*_3_ are the probabilities when the SWB is 1, 2, and 3, respectively. Maximum likelihood estimation methods can be used to conduct regression estimation to the SWB model of the Chinese rural elderly living independently. The results of the parameter estimation are presented in Table 2. The p-values of the deviance and Pearson criteria that determined the goodness of fit of the model were 0.9095 and 0.3991, respectively, and both were over 0.05. Therefore, the goodness of fit of the model was relatively good.

**Table 2:** **Regression results of the SWB model of the Chinese rural empty-nest elderly**

**Variables**	**Parameter estimation**	***p*** **-value**	**OR**
Intercept	1.4929	<0.001	
Age			
75–85 years old	−0.0103	0.9695	0.990
85–95 years old	−0.6238	0.0652	0.536
Gender			
Female	0.5842	0.0176	1.794
Education	−0.4328	0.3960	0.649
High school Middle school	0.8780	0.0387	2.406
Marriage			
Married and have spouse	0.2714	0.3291	1.312
Self-rated health condition			
Good	0.5767	0.0901	1.780
Fair	0.0355	0.8951	1.036
Number of children			
0	−0.3134	0.0561	0.731
1–2	0.0775	0.0773	1.081
Income			
Not good	−0.3915	0.0640	0.676
Fair	0.0696	0.8555	1.072
Frequency of meeting with children			
Rare	−0.0551	0.0665	0.9464
Sometimes	0.3173	0.0100	1.373

The parameter estimation was significant at the 10% significance level in terms of the impact factors of the SWB of the Chinese rural elderly living independently, as indicated in Table 2. The explanatory power was relatively strong.

The absolute value of the parameter estimation represents the impact degree of each impact factor on the SWB of the Chinese rural elderly living independently. Moreover, the symbol of the parameter estimation decides the impact direction of each impact factor on its corresponding SWB. The positive values represent the positive effect, and the negative values represent the negative effects. Oldest-old, high school education, no children in family, poor income, and rarely meet their children had negative impacts on their SWB. The remaining factors had positive impacts on the SWB of the Chinese rural elderly living independently.

The greater the OR value is, the greater the promotion effect on the SWB of the Chinese rural elderly living independently. The OR values of female, middle school education, married and have a spouse, good health condition, have one to two children, fair income, and sometimes meet their children were all above one. This result indicates the great promotion impact on the corresponding SWB. Moreover, the SWB of the rural elderly living independently with high school education was low and the feeling of unhappiness was strong. People with high education had high expectations of the future. The SWB was established on the gap between the real results and the subjective expectations. The greater the psychological pressure is, the more negative the impact on the SWB.

## Discussion

The number of rural elderly living independently is rapidly increasing. The elderly have become part of the vulnerable group in the society as they experience the gradual loss of physical and intellectual resources as well as a variety of chronic diseases. The rural elderly living independently are weaker than the urban elderly living independently in the vulnerable group.

This study finds that the scores of the Chinese rural elderly living independently in terms of PA, PE, and total SWB are relatively low and that the scores in the aspects of NA and NE are high. Their SWB has statistically significant differences in terms of gender, age, marriage, education, self-rated health condition, number of children, income, and relationship with children. For example, the SWB declines with age, and the SWB of females is higher than that of males. The elderly living independently who are married and have spouses, have children and frequently meet their children, and have a good income and self-rated health condition reported high scores of SWB. Good self-rated health indicate they rarely suffered from the chronic diseases and good income guarantee their easy utilization to health services [42]. In terms of education, the SWB of the elderly with middle school education is better than that of the elderly with primary school education. However, the elderly with high school education have the lowest SWB.

Further analysis on the main impact factors of the SWB of the Chinese rural elderly living independently shows that the factors (i.e., ages, gender, education, self-rated health condition, number of children, and frequency meeting children) have certain impacts on the SWB. Among these factors, oldest age, high school education, no children at home, poor income, and rarely meet their children have a negative impact on their SWB. The remaining factors have a positive impact.

The idea of raising children so the elderly will have someone to take care of them in their old age is ingrained in Chinese rural areas. For Chinese, the household environment and traditional cultures still affect Chinese people [43]. Traditional living in their twilight years with their family is the only way for the Chinese rural elderly. Children have an irreplaceable position in the eyes of the elderly, especially in terms of spiritual solace and life care. But now Chins’ family household is changing from a larger unit to smaller size. The living arrangements of the elderly are likely to live independently, which also cause the substantial changes of the co-residence of the elderly and their children [44].

There are studies found that though the elderly did not live with their family members, their relationship can affect their life quality [45]. According to family theory, the elderly who have stepped into the empty-nest period lose the social support and emotional communication because of the absence of their children. And the stronger emotional cohesion with the children can strengthen the well-being of the elderly. They encounter a new family, social problems, and individual health problems. The elderly living independently feel lonely and isolated because of the lack of communication with their children. They may express doubts regarding their existence and easily fall into a boring, helpless state. The long-term existence of these negative emotions can lead to the empty-nest syndrome, which results in the decline of the SWB.

Therefore we would like to propose some improve suggestions. *First,* the phenomenon of the rural elderly living independently should be alleviated. The children’s awareness of raising the elderly in the family should be strengthened. Publicity and education work on respecting and raising the elderly should be launched extensively in the rural areas. The responsibility of giving economic support, life care, and spiritual consolation to their parents should be emphasized to the children. The children should bear the responsibility of economically and emotionally supporting the elderly to ensure that the elderly lead a life of relative quality. The basic pension insurance system in rural areas should also be improved to ensure that the elderly living independently enjoy pension subsidies. More economic support can help increase the SWB so the elderly can avoid poverty and lead a relatively good life [46].

*Second,* the community function in rural areas should be tested. Special care should be provided to the elderly living independently. The traditional virtues in rural areas should be carried forward. The neighbors and friends of the elderly living independently should offer some basic community care services, such as the doing the housework and cooking, among others. Professional workers (e.g., doctors, social workers) can be included into the service system so that more professional services can be offered to the empty-nest elderly.

*Third,* the mental life of the Chinese rural elderly living independently should be given attention. The public should be mobilized and organized to volunteer in the ranks of empty-nest services and to fulfill the role of volunteers in the improvement of the rural elderly living independently. The construction of rural community cultural sports facilities should also be promoted to provide places where the elderly can conduct social activities. The community should hold sports activities that will be well received by the empty-nest elderly.

This study has several limitations. *First,* it takes the rural elderly living independently in Wenzhou City as the research objects. Wenzhou is one of the developed regions in China that benefits from advanced manufacturing. This study holds a certain reference value for the other developed regions in China. The regions where the economy is relatively backward, especially those in Midwestern China, still need further research. *Second,* the elderly living independently are a large vulnerable group. Further tracking studies should focus on their SWB to influence the improvement of decision making and government work.

## Conclusions

Many Chinese elderly increasingly face the serious problem of the "empty nest" phenomenon. However few studies concerned about the SWB of the elderly living independently. This study employs MUNSH to explore the SWB of the elderly living independently in rural areas of Wenzhou, a relatively developed region in China. Results indicate that age, female, low education, poor health condition and income and divorce are negative impact factors. But the children’s support can help to improve the SWB. The elderly living independently in rural Wenzhou, China have unfavorable SWB. Therefore it is suggested that we should strengthen the children’s awareness of raising the elderly, test the community function and highlight the importance of concerning about the mental life of the Chinese rural elderly living independently.
